# Monthly Summary

**Published:** 1873-05

**Authors:** 


### MONTHLY SUMMARY.

*Application for Chilblains.*—F. Rhien recommends an aqueous solution of iodine and tannin as a remedy for chilblains. He says that the result exceeded his expectations, five applications of the remedy being successful. The application has also been tried by others, with good results when properly applied. The solution is made as follows: about an ounce of tannin is dissolved in half a pint of water; seventy-four grains of iodine are dissolved in an ounce and three-fourths of spirits of wine; the two solutions are then mixed, and enough water is added to make up the whole to two and a half pints. The remedy is applied once daily, the best time being before going to bed. The mixture is gently warmed over a very slow fire; the affected part (*e. g.*, the hand) is dipped in it while still cold, and held there until the liquid, on being stirred, feels uncomfortably hot. The vessel is then removed from the fire, and the hand is dried over it, without gloves. The vessel used must be of earthenware or porcelain, not of metal. Care should be taken not to use too great a quantity of iodine, especially when abrasions are present. According to Rhien, four or five applications are sufficient.—*Apotheker Zeitung.*

---

*Chromic Acid.*—Dr. J. Dougall supports warmly the value of chromic acid (anhydride) as an antiseptic, disinfectant, and germ-preventive. Its coagulating power is about 10 times that of carbolic acid, 15 times that of nitric acid, 20 times that of corrosive sublimate, and 150 times that of chloralum. Its reaction with gelatine is as delicate as that of tannic acid, giving a response with 1 in 5,000. Chromic acid is well adapted for estimating albumen volumetrically, thus: Fill a wide-mouthed burette to a multiple of 100 with the albuminous liquid (say urine,) add solution of chromic acid (4 grs. to ℥i) in slight excess; shake, set aside for 24 hours and read off. Carbolic acid does not combine with ammonia or sulphuretted hydrogen; chromic acid does. *Lancet.*

---

*Tea vs. Brandy.*—The question of replacing brandy by an allowance of tea is occuping the serious attention of the Russian military authorities. According to the *Viedomosti*, of St. Petersburg, the Minister of War has submitted the question to the deliberation of "Specialists," who, better than any one else, can appreciate the utility and the results produced by brandy and tea. The general opinion in military circles is strongly in favor of the use of tea; but it appears to be doubted whether it can take the place of brandy without causing too great an excess in the budget set down for the maintenance of the troops. It results from calculations made by Dr. Steinberg that an annual ration of tea and sugar would cost 322 roubles 15 copecks for every 100 soldiers, while a ration of brandy costs only 30 roubles 60 copecks; the difference being 282 roubles 55 copecks. The pound of tea is estimated at four francs; and the Russian army numbers, as we have recently been told, about 1,500,000 men on peace footing; it is therefore highly probable that the military authorities will take some time to consider the advisability of adopting such a costly change.—*Med. & Surg. Reporter.*

---

*Carbolic Acid as a Local Anæsthetic in Boils, Whitlows, etc.*—Dr. J. N. Taylor writes to the *Nashville Medical Journal*:

"I have frequently used carbolic acid in minor surgery, but not until recently to my great satisfaction.

"About the middle of November of last year I was the subject of a very large and painful boil, on the ulna side of the forearm. I saturated a piece of worn linen in an aqueous solution of carbolic acid, and applied until all sensibility of the part had ceased; then, with a piece of ordinary writing paper, twisted to a sharp point, and dipped in a solution of the acid (full strength,) marked out the line of the desired incision; then, with an ordinary scalpel, I made a free incision, fully an inch in length, without any pain whatever.

"I greatly prefer the application of the acid thus used to the freezing process."

---

*Danger of Morphine Injections, with Chloroform Inhalations.*—A good deal has been written about the union of morphine injections with chloroform inhalatious to produce prolonged surgical operations. M. Demarquay concludes, from a short series of experiments, that it is a method liable to especial dangers, arising from lowering of the temperature.—*Med. Record.*

---

*Singular Causes of Death.*—The last publication of the British death rate and its causes is curious reading. One man died from the bite of a cat; and two more from the bites respectively of a ferret and an adder. Another was stung to death by bees. A man and a boy died of falling from velocipedes, and and an old lady was killed by injuries inflicted by that agreeable machine. The swallowing of a shell, a screw and a cherry-stone, put a period to the lives of three infants, while two died of putting one a stone, the other a bead into the ear. Swallowing bones sent three people out of the world, swallowing coins finished two, and swallowing a pin quickly pricked on grim Death for one. A scratch from a thorn killed a woman of middle age; improper medicine poisoned eight people, and improper food five; 444 young children were smothered by bed-clothes, and 930 persons during the year lost their lives in railway accidents. The proportion of suicides to every million of the population is about 70, the deaths by hanging, the knife and drowning being most numerous. Heart disease the year's record shows to be increasing; a state of things which is said by eminent physicians to be caused by the greater wear and tear of business and the increased mental activity of the age.—*Med. & Surg. Reporter.*

---

*Anodyne Colloid.*—Dr. H. M. Lackersteen gives (*Brit. Med. Journal*) the following formula for a topical anodyne colloid which he has found useful in neuralgia, sciatica, lumbago, all muscular pains, etc. It relieves local pains for the time, and procures a good night's rest. ℞.—Hydride of amyl, ℥j. aconitia, gr. j; veratria, gr. vj; ethereal collodion to ℥ ij. The amyl, by its rapid volatilization, often produces, almost instantaneously, the desired result: but, should the pain continue the alkaloids can be brought into activity by applying a piece of moist spongiopiline over the collodion film. The amyl hydrid is the only new ingredient: but I think the colloid is a clean and elegant preparation.—*Med, News.*

---

*Hops as an External Preparation.*—If possessed of any virtue as an anodyne, and popularly hops have great reputation as such, the effect is best obtained by *steaming* a muslin bag full of them, on the side to be applied to the body, until quite soft and damp—not wet.

The ordinary method of wringing out in hot water wastes all the strength of the hops, and makes a nasty mess.—(*Druggists' Circular.*)

